# Effects of exercise on pulse wave velocity in hypertensive and prehypertensive patients: a systematic review and meta-analysis of randomized controlled trials

**DOI:** 10.3389/fcvm.2025.1504632

**Published:** 2025-02-17

**Authors:** Haoyu Xi, Liwen Du, Gen Li, Shiyan Zhang, Xiang Li, Yuanyuan Lv, Lin Feng, Laikang Yu

**Affiliations:** ^1^ Beijing Key Laboratory of Sports Performance and Skill Assessment, Beijing Sport University, Beijing, China; ^2^ Department of Strength and Conditioning Assessment and Monitoring, Beijing Sport University, Beijing, China; ^3^ School of Physical Education & Sports Science, South China Normal University, Guangzhou, China; ^4^ School of Sport Sciences, Beijing Sport University, Beijing, China; ^5^ China Institute of Sport and Health Science, Beijing Sport University, Beijing, China; ^6^ Beijing Sports Nutrition Engineering Research Center, Beijing, China

**Keywords:** exercise, pulse wave velocity, arterial stiffness, hypertension, prehypertension, systematic review, meta-analysis

## Abstract

**Background:**

This study aimed to examine the effects of exercise on pulse wave velocity (PWV) in hypertensive and prehypertensive patients.

**Methods:**

A comprehensive search was performed in PubMed, Cochrane, Embase, Scopus, and Web of Science, covering data up to August 31, 2023. A meta-analysis was conducted to determine the weighted mean difference (WMD) and 95% confidence interval for the effects of exercise on PWV in hypertensive and prehypertensive patients.

**Results:**

A total of 17 studies met the inclusion criteria. Exercise had a significant effect on improving PWV in hypertensive and prehypertensive patients (WMD, −0.93, *P* = 0.0001). Subgroup analysis showed that aerobic exercise (WMD, −1.29, *P* = 0.0004) significantly improved PWV in hypertensive and prehypertensive patients. Moreover, aerobic exercise, specifically moderate-intensity aerobic exercise (WMD, −1.69, *P* = 0.03), conducted for ≥12 weeks (WMD, −1.54, *P* = 0.002), ≥3 times per week (WMD, −1.44, *P* = 0.002), ≤60 min per session (WMD, −1.50, *P* = 0.02), and ≥180 min per week (WMD, −0.57, *P* = 0.005), was more effective in improving PWV in hypertensive and prehypertensive patients, especially in middle-aged individuals (WMD, −1.78, *P* < 0.0001).

**Conclusion:**

To improve arterial stiffness, hypertensive and prehypertensive patients, particularly middle-aged individuals, are recommended to participate in a minimum of 12 weeks of moderate-intensity aerobic exercise at least 3 times per week for less than 60 min per session, with a goal of 180 min per week being achieved by increasing the frequency of exercise.

**Systematic Review Registration:**

https://www.crd.york.ac.uk/prospero/display_record.php?RecordID=458981, identifier: CRD42023458981.

## Introduction

Hypertension is a chronic non-communicable disease that poses a significant threat to human health and serves as one of the major factors contributing to cardiovascular complications, leading to over 10 million deaths globally each year (1). The prevalence of hypertension is generally high worldwide, with adult males and females exhibiting rates of 24% and 20%, respectively. Hypertension is also referred to as the “silent killer” due to its often asymptomatic nature (2). Failure to treat hypertension in a timely manner, can result in serious complications such as heart failure, renal failure, hypertensive retinopathy, and atherosclerosis (3). Furthermore, patients with hypertension are at a higher risk of developing chronic diseases (e.g., type 2 diabetes mellitus and hyperlipidaemia) compared to the general population (4). Atherosclerosis is a chronic degenerative disease that affects the inner walls of the arteries, causing damage and progressive stiffness over a prolong period (5). Given the impact of atherosclerosis on hypertensive patients, it is crucial to diagnose them to detect and prevent atherosclerosis. Pulse wave velocity (PWV) measurement is a non-invasive and painless diagnostic test that indicates the conduction velocity of the aortic pulse wave, which is strongly correlated with atherosclerosis (6).

PWV is considered to be the simplest, most widely used, and most recognized technique for measuring arterial stiffness (7, 8). The PWV measurement involves comparing the difference between two recording points on the pulse moving line and the delay between corresponding points on the wave (pressure or flow) that are unaffected by wave reflection (8). In large central elastic arteries, such as the aorta, PWV increases significantly with age, whereas PWV in arteries of the upper extremities remains stable (9). Among the various PWV measurements, the carotid-femoral PWV (cfPWV) has been the most extensively studied, and it is used in various atherosclerosis studies conducted by research centers in Europe (10), Australia (11), and the United States (12). In East Asian countries such as Japan, brachial-ankle PWV (baPWV) is widely adopted for assessing arterial stiffness (13–15).

The prevalence of hypertension rises with age (16). Maintaining a healthy lifestyle and regular exercise can effectively reduce the morbidity and mortality rates associated with hypertension, making it crucial for the proper treatment and prevention of hypertension (17, 18). Aerobic exercise is recommended for the prevention and treatment of hypertension, and studies have found that it can lower systolic blood pressure by 10 mmHg and diastolic blood pressure by 7 mmHg in hypertensive patients (19).

However, variations in training parameters such as frequency, intensity, duration, and type of exercise intervention can exert varying degrees of impact on atherosclerosis (20–22). Numerous studies have reported that single, moderate- to high-intensity aerobic exercise can improve arterial stiffness. Kingwell et al. (22) showed increased aortic blood flow and carotid artery pressure, along with decreased aortic conduction velocity, after a 30 min cycling session at 65% of maximal oxygen uptake (VO_2_max), indicating that acute sustained aerobic exercise reduces pulse-wave conduction velocity, thereby enhancing systemic arterial compliance.

Despite the belief that exercise can improve arterial stiffness to some extent in hypertensive and prehypertensive patients, previous studies have not focused on specific exercise modalities, resulting in substantial heterogeneity among interventions. Significant differences exist in the physiological changes induced by different exercise modes. Consequently, the primary aim of this systematic review and meta-analysis was to investigate the effects of exercise on PWV in hypertensive and prehypertensive patients. The secondary objectives were to explore the optimal type of exercise, intervention duration, intensity, frequency, session duration, and weekly time in hypertensive and prehypertensive patients.

## Methods

### Design

The Cochrane Selection Manual and the Preferred Reporting Items for Systematic Reviews and Meta-Analysis guidelines were rigorously adhered for conducting this systematic review and meta-analysis (23). The protocol was registered on PROSPERO with, registration number: CRD42023458981.

### Search strategy

A comprehensive search was conducted in PubMed, Cochrane Library, Embase, Scopus, and Web of Science database to retrieve relevant articles published up to August 31, 2023. The search strategy encompassed the following keywords and MESH: exercise, pulse wave velocity, and hypertension. In addition, a manual search was conducted through the reference lists of all identified studies, including reviews and meta-analyses, to identify potentially eligible studies. This screening process was independently undertaken by two authors (HX and LD) and any disagreements were resolved through discussions with the third author (LY).

### Inclusion and exclusion criteria

The inclusion criteria for this study were: (1) type of study: randomized controlled trials (RCTs) with human subjects; (2) study intervention: exercise as the primary or sole intervention, without limitations on the type, intensity, frequency, duration, or total amount of exercise; (3) including participants who were hypertensive or prehypertensive, defined as having a systolic BP of 140 mmHg or greater, a diastolic blood pressure of 90 mmHg or higher, or systolic BP between 120 and 139 mmHg or diastolic BP between 80 and 89 mmHg, respectively, according to World Health Organization's criteria; (4) study outcome: PWV as the primary or secondary outcome indicator.

The exclusion criteria were as follows: (1) article type: reviews and conference publications; (2) control group: lack of an appropriate control group; (3) data completeness: insufficient data or information, such as the lack of for reporting specific types of exercise interventions or the absence of raw data.

### Data extraction

Two authors (HX and LD) independently reviewed and extracted data for each study, including the name of the first author, publication year, sample size, intervention characteristics (type of intervention, intervention duration, frequency, intensity, session duration), participant characteristics (age), and the mean and standard deviation (SD) values reflecting the change in PWV following the intervention.

### Methodological quality assessment

The Cochrane Collaboration tool (RoB2) and Physiotherapy Evidence Database (PEDro) Scale were used to assess the risk of bias and the quality of the included studies, respectively (24, 25). RoB2 was based on selection bias, performance bias, detection bias, attrition bias, reporting bias, and other biases. Specifically designed for evaluating the quality of RCTs in physical therapy studies, the PEDro Scale comprises 11-item. The total PEDro scores for RCTs ranged from 0 to 10. Scores exceeding 9 are deemed excellent, while scores between 6 and 8 are considered good, 4 to 5 average, and below 4 are regarded as poor quality (26, 27). Two authors (HX and LD) independently conducted the methodological quality assessment, and any discrepancies were resolved through discussions with the third author (LY).

### Statistical analysis

We calculated the change in mean and SD values of PWV and summarized the data using a random-effects model to derive weighted mean difference (WMD) and 95% confidence interval (CI). If there was a high level of heterogeneity (*I*^2^ > 60%), meta-regression, subgroup analysis, and sensitivity analysis were conducted to interpret the results (28).

In subgroup analyses, we explored the effects of various factors on PWV, including the type of exercise (aerobic exercise, resistance exercise, multicomponent training), duration of aerobic exercise (<12 weeks, ≥12 weeks), intensity (moderate-intensity, vigorous-intensity) (29), frequency (<3 times per week, ≥3 times per week), session duration (≤60 min per session, >60 min per session), weekly time (<180 min per week, ≥180 min per week), and participants' age (middle-aged, 45 ≤ age < 60; older adult, ≥60). The forest plots were generated using RevMan. 5 software and meta-regression, funnel plot, and sensitivity analysis were performed using Stata software. A *P* < 0.05 was considered statistically significant.

## Results

### Study selection

**Figure 1:**
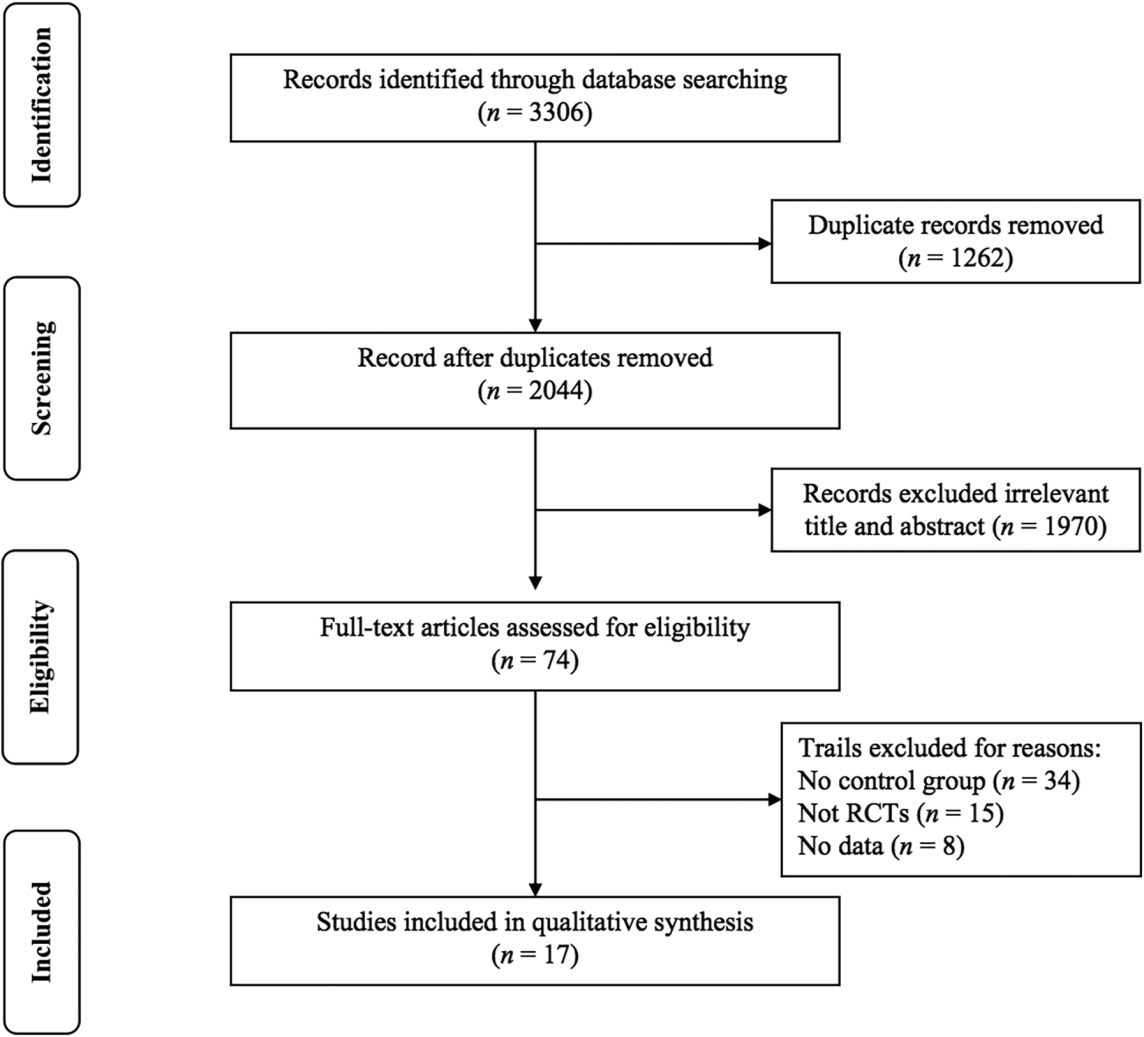
PRISMA flowchart of study selection.

As shown in Figure 1, an initial search of the database yielded a total of 3,306 articles, while 17 articles were retrieved from other sources. After eliminating duplicates, a total of 1,856 studies remained. Following a screening of titles and abstracts, 74 potentially eligible studies were identified. Finally, upon reviewing the full texts, 17 studies (30–46) examining the effects of exercise on PWV in hypertensive and prehypertensive patients were deemed suitable for systematic review and meta-analysis.

### Characteristics of the included studies

As shown in Table 1, this study included a total of 17 studies (30–46) on the effects of exercise on PWV in hypertensive and prehypertensive patients, published between 2005 and 2022. The overall sample size comprised 940 hypertensive and prehypertensive patients, with 472 allocated to the exercise intervention group and 468 to the control group. Of these, 14 studies (30, 32–39, 42–46) focused on the hypertensive population and 3 studies (31, 40, 41) covered the prehypertensive population. The studies varied in terms of subject ages, exercise type, duration, frequency, intensity, session duration, and weekly time. Among the interventions, 9 studies (30, 32, 34, 36, 38, 42, 44–46) used aerobic exercise, 5 studies (31, 35, 40, 41, 43) used multicomponent training, and 2 studies (37, 39) used resistance exercise. The duration of the exercise interventions ranged from 2 to 26 weeks, averaging 14.69 weeks. The frequency of interventions ranged from 2 to 4 times per week, averaging 2.75 times per week. The session duration ranged from 6 to 80 min, averaging 50.28 min. The weekly time ranged from 9 to 180 min, averaging 72.44 min. Five studies (34, 36, 38, 44, 45) utilized moderate-intensity for the aerobic exercise intervention, while only 2 studies (30, 42) opted for vigorous-intensity.

**Table 1:** Characteristics of the studies included in this meta-analysis.

Study	Sample size (M/F)	Mean age (y)	Intervention	Minutes per session (min)	Frequency (times/week)	Minutes per week (min)	Duration (weeks)	Results on PWV
Aghaei et al. (30)	30 (30/0)	43 to 53.8	HIIT	SDHIIT: 29 LDHIIT: 47	3	SDHIIT: 87 LDHIIT: 141	8	baPWV decline
Beck et al. (31)	58 (39/19)	18 to 35	Combined	60	3	180	8	cfPWV decline
Fetter et al. (32)	33 (0/33)	45 to 68	Yoga Stretching	75	2	150	12	cfPWV decline
Figueroa et al. (33)	25 (0/25)	55.5 to 56.4	WVET	NR	3	NR	12	baPWV decline
Guimarães et al. (34)	65 (23/42)	36 to 58	Running RE	80	3	240	16	cfPWV decline
Lee et al. (35)	20 (0/20)	70 ± 4	Taekwondo	60	3	180	12	baPWV decline
Madden et al. (36)	52 (30/22)	69.3 ± 0.6	AE	60	3	180	24	cfPWV decline
Miura et al. (37)	221	60 to 88	RE	40	2	80	12	baPWV decline
Pascoalino et al. (38)	40 (28/12)	45 ± 6	Running	50	1	50	12	No change
Rodrigues et al. (39)	33 (11/22)	57–63	IHT	8	3	24	12	cfPWV decline
Son et al. (40)	40 (0/40)	15 ± 1	RE Badminton	60	3	180	12	baPWV decline
Songcharern et al. (41)	30 (30/0)	18 to 22	Combined	60	3	180	8	No change
Sosner et al. (42)	42 (22/20)	65 ± 7	MICT HIIT	MICT:34 HIIT:30	3	MICT:102 HIIT:90	2	cfPWV decline
Stewart et al. (43)	82 (38/44)	55 to 75	Running	NR	NR	NR	NR	No change
Wong et al. (44)	41 (0/41)	49 to 67	Stair climbing	NR	NR	NR	12	baPWV decline
Wong et al. (45)	100 (0/100)	67 to 85	Swimming	1–5 week: 25–30 6–20 week:40–45	3–4	1–5 week:60–120 6–20 week:120–180	20	crPWV decline
Wong et al. (46)	28 (0/28)	19 to 27	Pilates training	60	3	180	12	baPWV decline

HIIT, high-intensity interval training; SD, short-duration; LD, long-duration; IHT, isometric handgrip training; WVET, whole-body vibration exercise training; AE, aerobic exercise; RE, resistance exercise; MICT, moderate-intensity continuous training; NR, no report; aoPWV, aortic pulse wave velocity; baPWV, brachial-ankle pulse wave velocity; cfPWV, carotid–femoral pulse wave velocity; crPWV, carotid-radial pulse wave velocity.

### Meta-analysis results

#### Effects of exercise on PWV in hypertensive and prehypertensive patients

**Figure 2:**
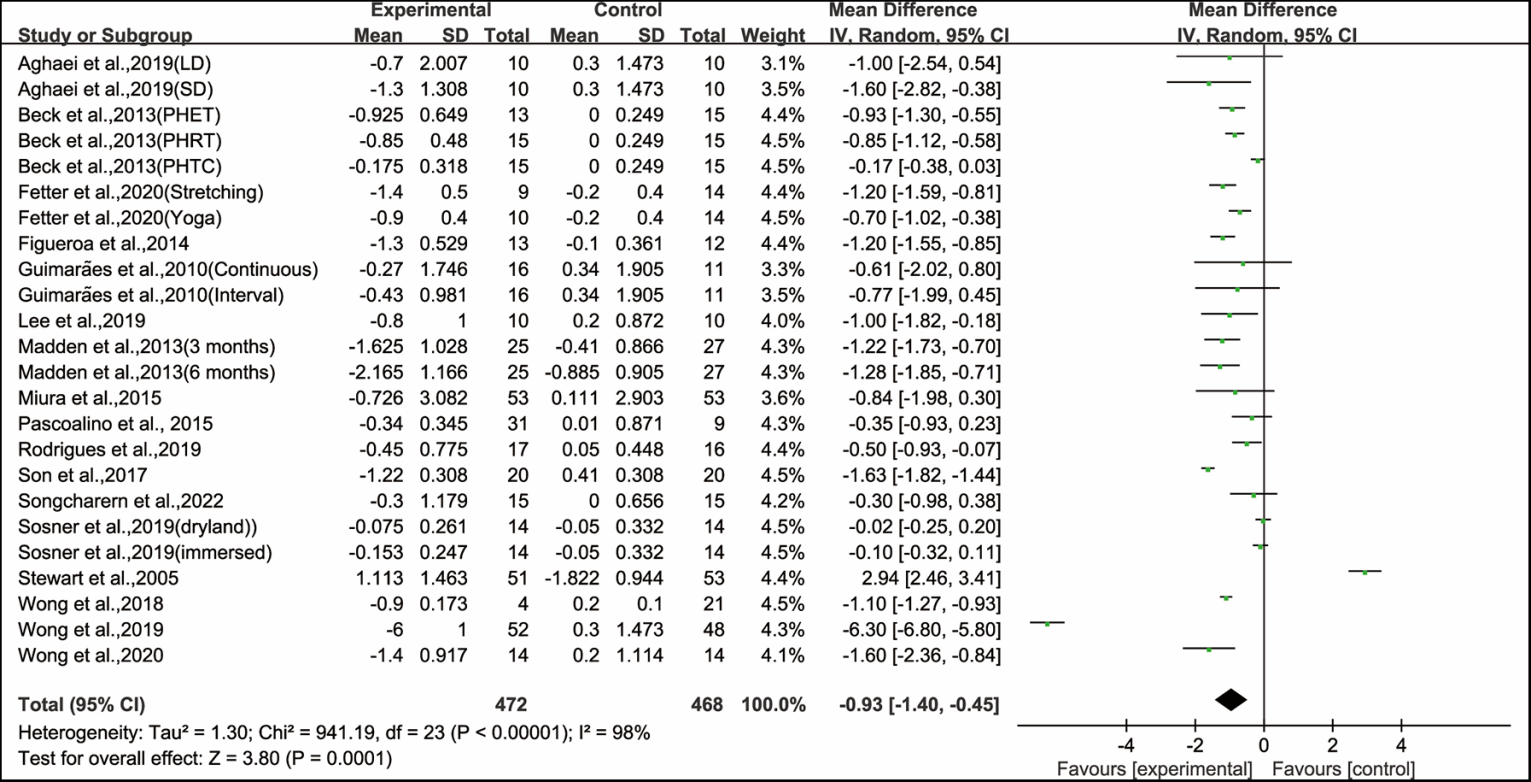
Meta-analysis results of the effects of exercise on PWV in hypertensive and prehypertensive patients.

After analysing the data from all the included studies, we found that exercise had a significant effect on improving PWV in hypertensive and prehypertensive patients (WMD, −0.93; 95% CI, −1.40 to −0.45, *P* = 0.0001, *I*^2^ = 98%, Figure 2).

Our meta-analysis results showed high heterogeneity in PWV (*I*^2^ = 98%), to explain the heterogeneity between included studies and find modifiable factors of exercise, meta-regression analysis, subgroup analysis, and sensitivity analysis were further performed.

### Meta-regression

Meta-regression analyses were performed on intervention duration, frequency, session duration, weekly time, subjects' age, basal systolic blood pressure (SBP), and basal diastolic blood pressure (DBP). There were no significant associations between session duration (*P* = 0.547), frequency (*P* = 0.879), weekly time (*P* = 0.170), intervention duration (*P* = 0.725), subjects' age (*P* = 0.610), SBP (*P* = 0.192), and DBP (*P* = 0.106) and PWV (Supplementary Figure S1).

### Subgroup analysis

**Figure 3:**
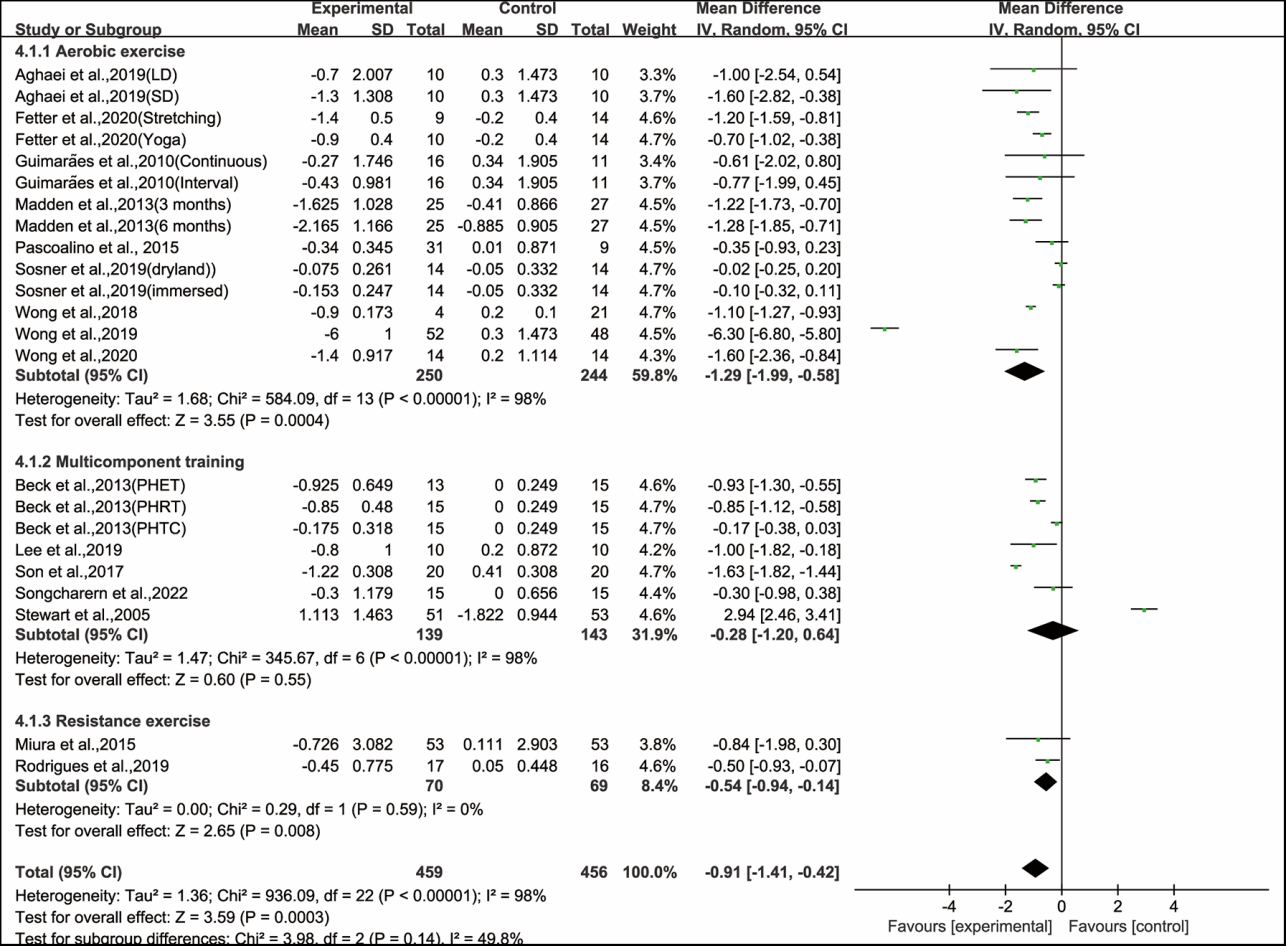
Meta-analysis results of the effects of types of intervention on PWV in hypertensive and prehypertensive patients.

Stratifying the analysis by types of exercise interventions, aerobic exercise (WMD, −1.29; 95% CI, −1.99 to −0.58, *P* = 0.0004, *I*^2^ = 98%) and resistance exercise (WMD, −0.28; 95% CI, −1.20 to −0.64, *P* = 0.008, *I*^2^ = 98%) significantly improved PWV, while multicomponent training had no significant effect on improving PWV in hypertensive and prehypertensive patients (WMD, −0.91; 95% CI, −1.41 to 0.42, *P* = 0.55, *I*^2^ = 0%, Figure 3). Since aerobic exercise was the most efficient intervention type, we conducted further subgroup analyses focusing on aerobic exercise.

**Figure 4:**
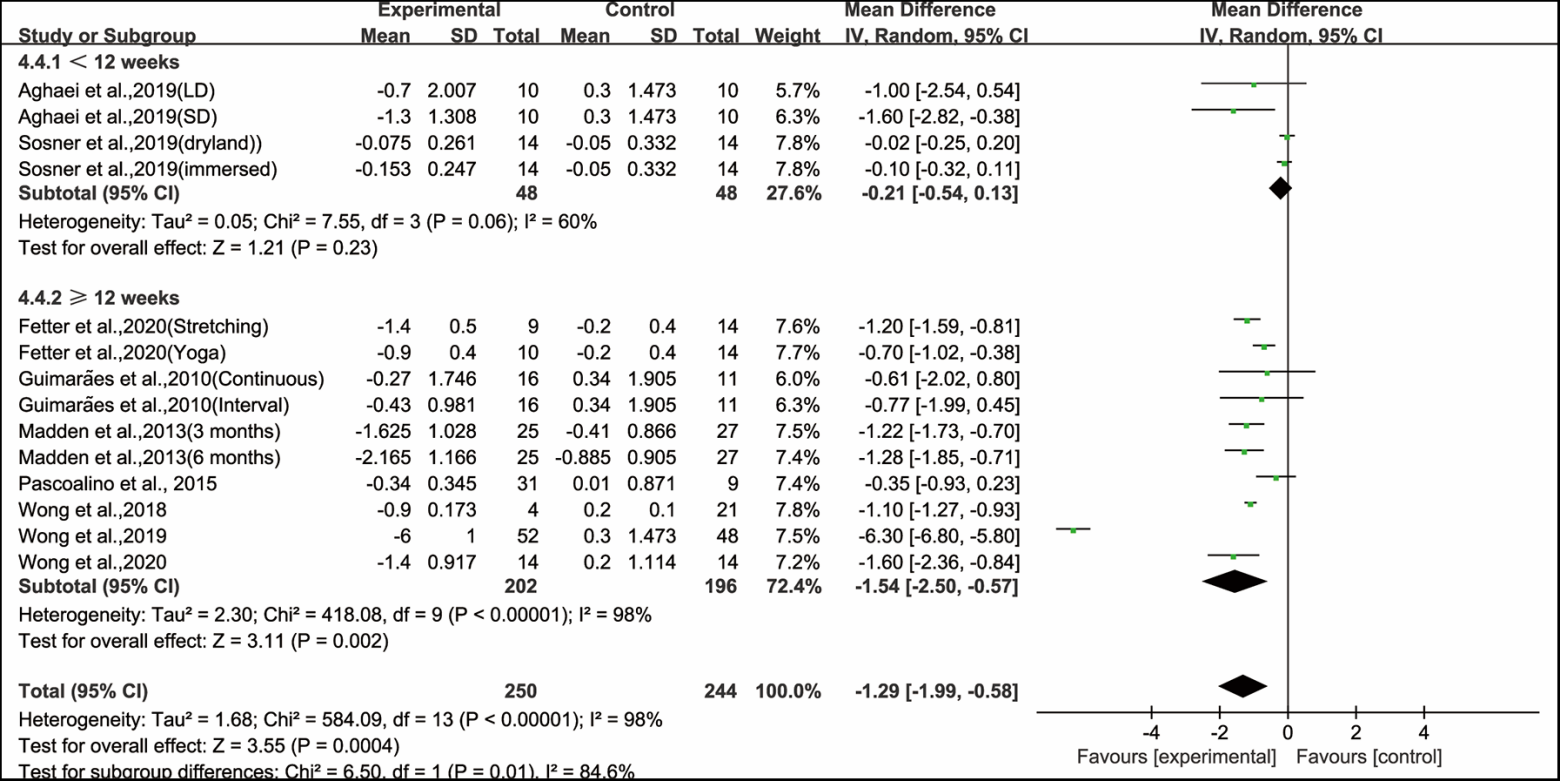
Meta-analysis results of the effects of aerobic exercise duration on PWV in hypertensive and prehypertensive patients.

When analyzing the subgroup by the intervention duration, ≥12 weeks of aerobic exercise significantly improved PWV (WMD, −1.54; 95% CI, −2.50 to −0.57, *P* = 0.002, *I*^2^ = 98%), while < 12 weeks of aerobic exercise had no significant effect on improving PWV in hypertensive and prehypertensive patients (WMD, −0.21; 95% CI, −0.54 to 0.13, *P* = 0.23, *I*^2^ = 60%, Figure 4).

**Figure 5:**
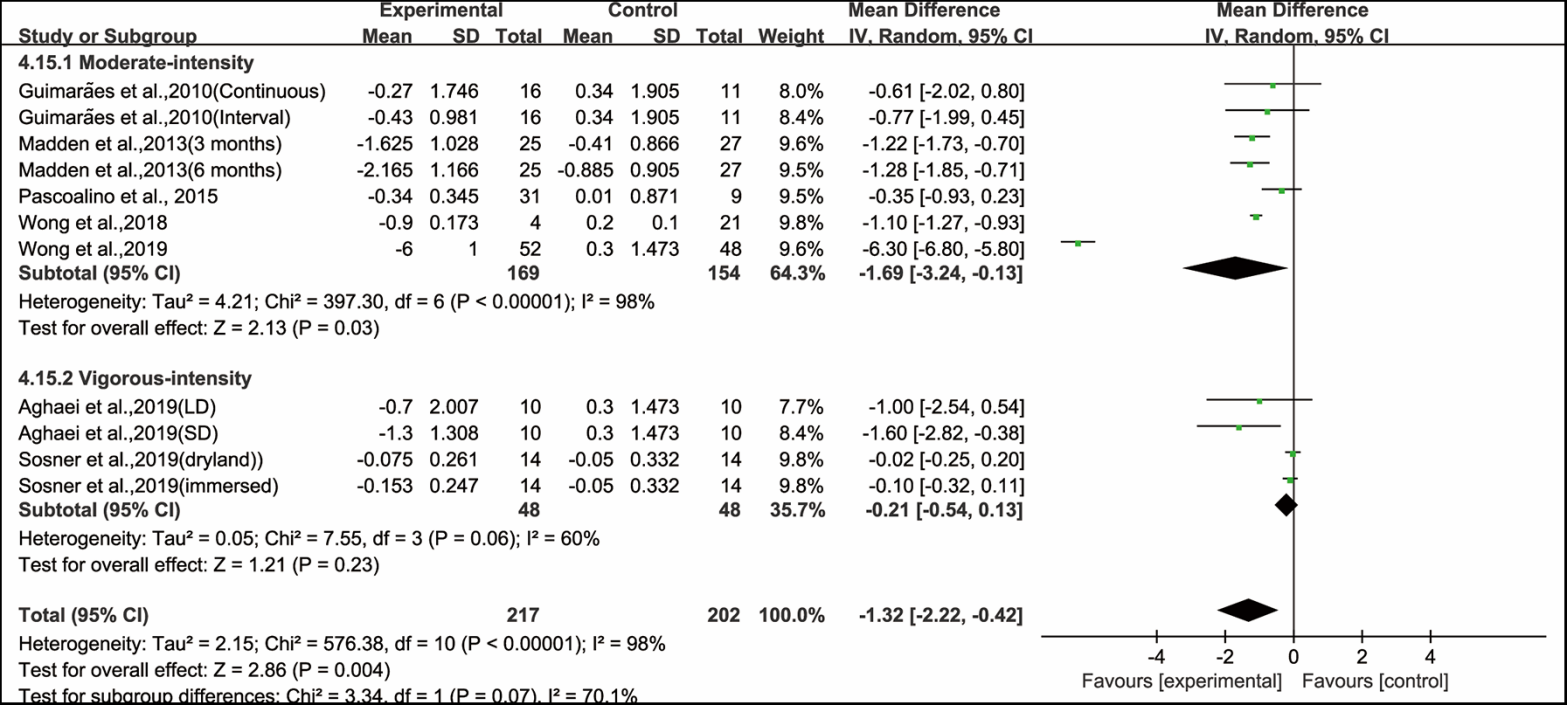
Meta-analysis results of the effects of intensity of aerobic exercise on PWV in hypertensive and prehypertensive patients.

In addition, when analyzing the subgroup by intensity, moderate-intensity aerobic exercise significantly improved PWV (WMD, −1.69; 95% CI, −3.24 to −0.13, *P* = 0.03, *I*^2^ = 98%), while vigorous-intensity aerobic exercise had no significant effect on improving PWV in hypertensive and prehypertensive patients (WMD, −0.21; 95% CI, −0.54 to 0.13, *P* = 0.23, *I*^2^ = 60%, Figure 5).

**Figure 6:**
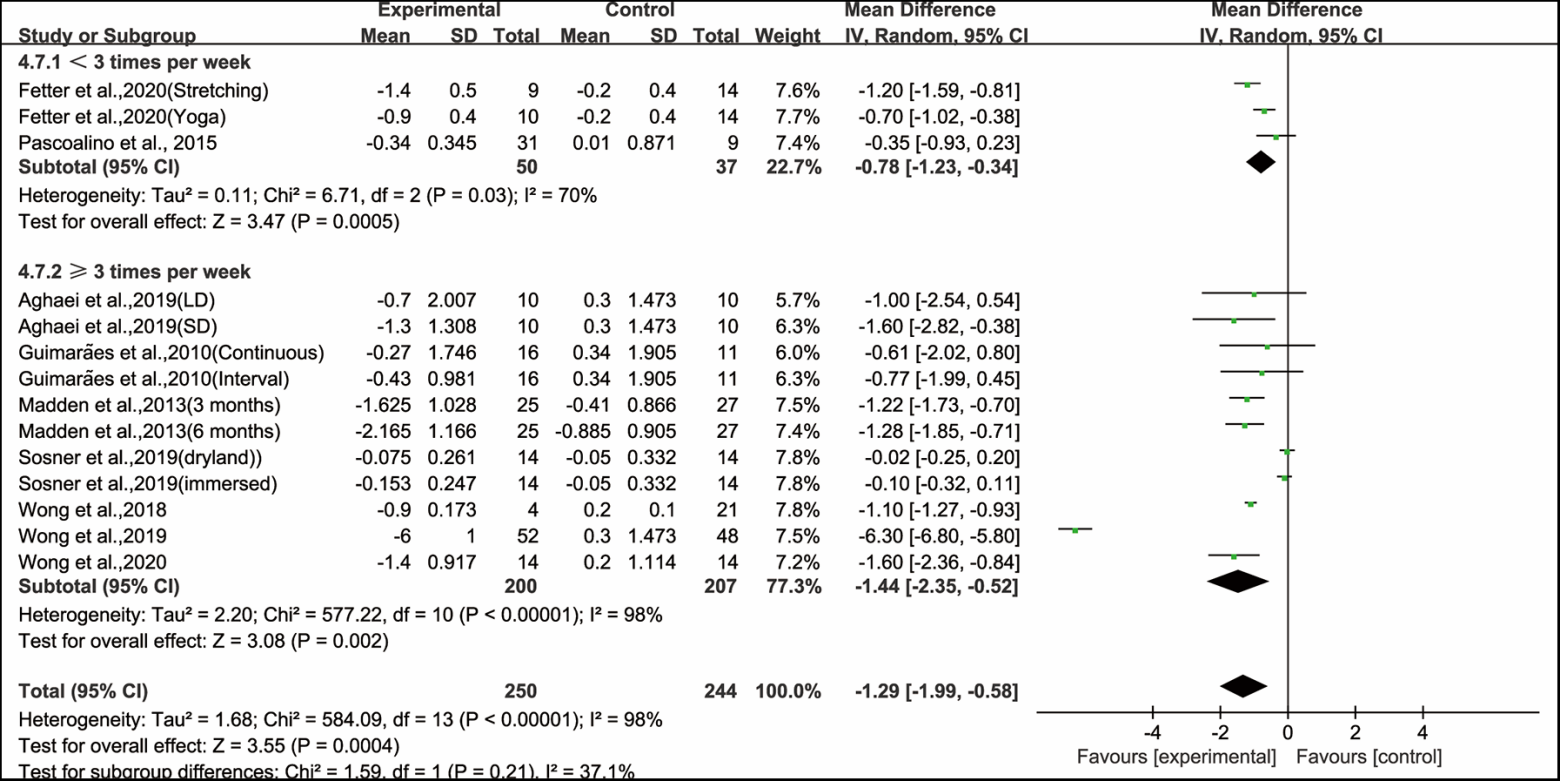
Meta-analysis results of the effects of frequency of aerobic exercise on PWV in hypertensive and prehypertensive patients.

Besides, when analyzing the subgroup by frequency, aerobic exercise conducted for <3 times per week (WMD, −0.78; 95% CI, −1.23 to −0.34, *P* = 0.0005, *I*^2^ = 70%) and ≥3 times per week significantly improved PWV in hypertensive and prehypertensive patients (WMD, −1.44; 95% CI, −2.35 to −0.52, *P* = 0.002, *I*^2^ = 98%, Figure 6). Specifically, aerobic exercise conducted for ≥3 times per week had a greater effect on improving PWV in hypertensive and prehypertensive patients.

**Figure 7:**
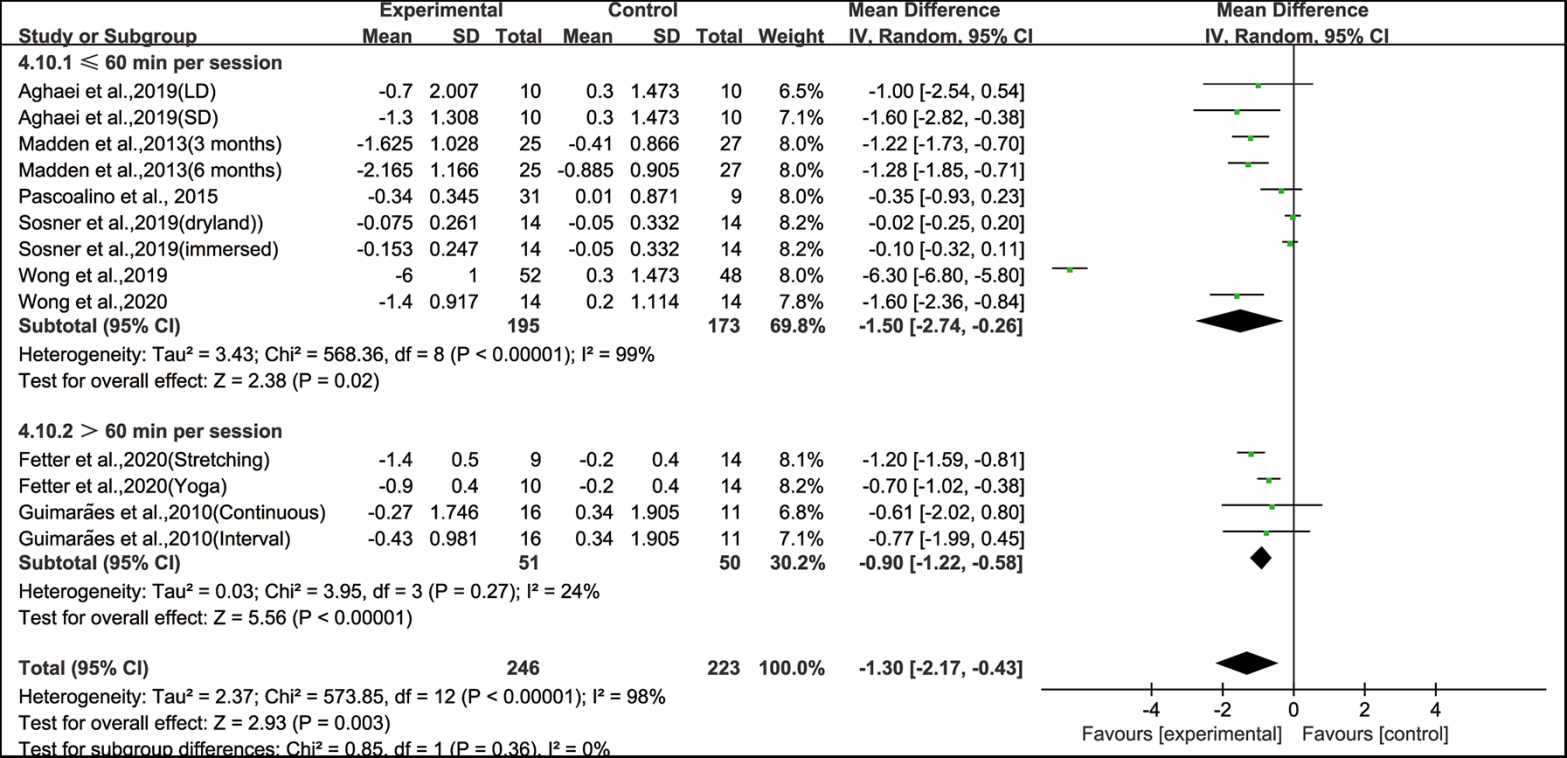
Meta-analysis results of the effects of duration of aerobic exercise per session on PWV in hypertensive and prehypertensive patients.

Furthermore, when analyzing the subgroup by session duration, aerobic exercise conducted for ≤60 min per session (WMD, −1.50; 95% CI, −2.74 to −0.26, *P* = 0.02, *I*^2^ = 99%) and >60 min per session significantly improved PWV in hypertensive and prehypertensive patients (WMD, −0.90; 95% CI, −1.22 to −0.58, *P* < 0.00001, *I*^2^ = 24%, Figure 7). Specifically, aerobic exercise conducted for ≤60 min per session had a greater effect on improving PWV in hypertensive and prehypertensive patients.

**Figure 8:**
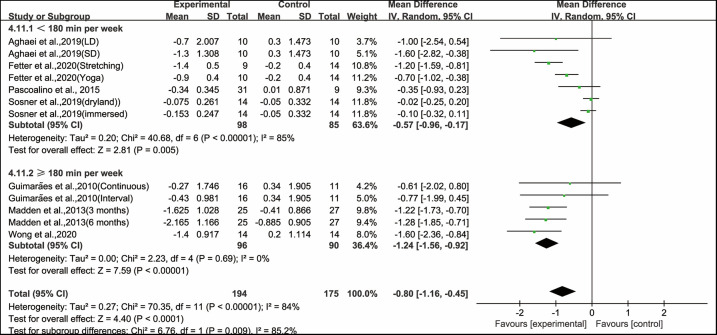
Meta-analysis results of the effects of duration of aerobic exercise per week on PWV in hypertensive and prehypertensive patients.

Moreover, when analyzing the subgroup by weekly time, aerobic exercise conducted for <180 min per week (WMD, −0.57; 95% CI, −0.96 to −0.17, *P* = 0.005, *I*^2^ = 85%) and ≥180 min per week significantly improved PWV in hypertensive and prehypertensive patients (WMD, −1.24; 95% CI, −1.56 to −0.92, *P* < 0.00001, *I*^2^ = 0%, Figure 8). Specifically, aerobic exercise conducted for ≥180 min per week had a greater effect on improving PWV in hypertensive and prehypertensive patients.

**Figure 9:**
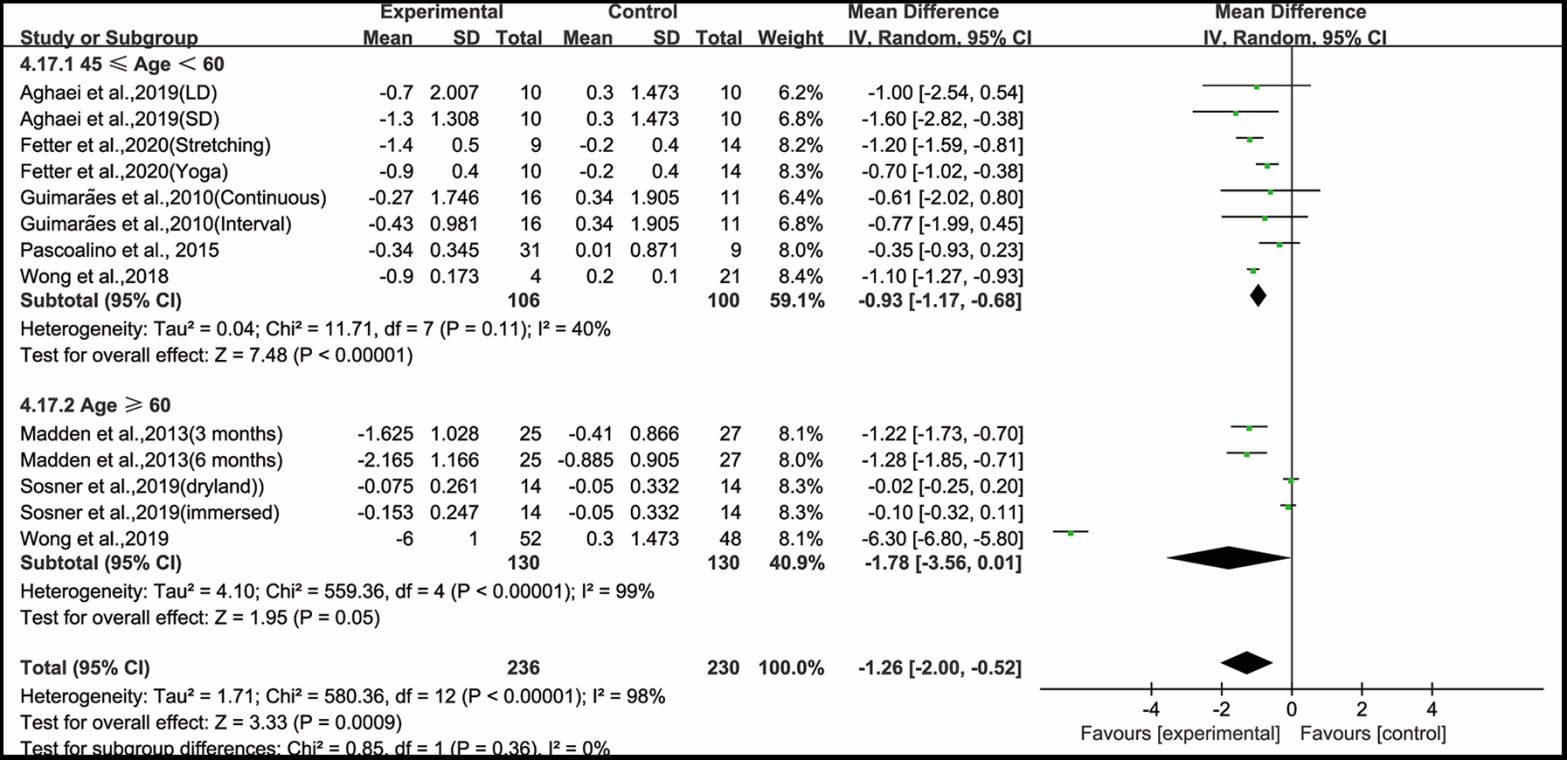
Meta-analysis results of the effects of aerobic exercise on PWV in middle-aged and older adult hypertensive and prehypertensive patients.

Finally, when analyzing the subgroup by subjects' age, aerobic exercise significantly improved PWV in middle-aged hypertensive and prehypertensive patients (WMD, −0.93; 95% CI, −1.17 to −0.68, *P* < 0.00001, *I*^2^ = 40%), while aerobic exercise had no significant effect on improving PWV in older adult hypertensive and prehypertensive patients (WMD, −1.78; 95% CI, −3.56 to 0.01, *P* = 0.05, *I*^2^ = 99%, Figure 9).

### Sensitivity analysis

Sensitivity analyses indicated that excluding any of the included studies did not alter the direction or magnitude of the overall effect of exercise on PWV in hypertensive and prehypertensive patients (Supplementary Figure S2).

### Risk of bias

To assess the methodological quality of the included studies and address these potential biases, we utilized the RoB2 tool. This tool allowed us to evaluate selection bias, performance bias, detection bias, attrition bias, reporting bias, and other biases comprehensively (Supplementary Figure S3). By employing this rigorous assessment, we were able to gain insights into the potential sources of bias within the included studies and take steps to mitigate their impact on our findings. Furthermore, according to the PEDro scale, which is a validated tool for assessing the methodological quality of studies in physiotherapy, among the 17 included studies, 5 were rated as excellent, and 12 studies were rated as good (Supplementary Table S1). This assessment provided additional confidence in the reliability and validity of the included studies.

### Publication bias

To assess potential publication bias, we employed two methods: visual inspection of funnel plots and Egger's test. Visual inspection of the funnel plot suggested the absence of funnel plot asymmetry, indicating no evidence of publication bias (Supplementary Figure S4). Additionally, based on the results of Egger's test, small sample size studies did not significantly influence the results of PWV (*P* = 0.712, Supplementary Table S2). These findings further support the robustness of our meta-analysis and the reliability of our conclusions.

## Discussion

The aim of this study was to explore the effects of exercise on PWV in hypertensive and prehypertensive patients. Out of the initial 3,306 search records, 17 studies were included, and the findings indicated that exercise, particularly aerobic and resistance exercises, significantly improved PWV in hypertensive and prehypertensive patients. Subgroup analyses revealed that aerobic exercise, specifically moderate-intensity aerobic exercise, conducted for ≥12 weeks, ≥3 times per week, ≤60 min per session, and ≥180 min per week, was more effective in improving PWV in hypertensive and prehypertensive patients, especially in middle-aged individuals.

### Effects of exercise on PWV in hypertensive and prehypertensive patients

This study suggested that exercise had the potential to improve arterial stiffness, such reducing PWV, in hypertensive and prehypertensive patients. Our findings indicated an overall reduction in PWV of 0.90 (WMD), which holds significant clinical implications for hypertensive and prehypertensive patients. It is noteworthy that under the umbrella of exercise interventions for hypertensive and prehypertensive patients, most interventions involve aerobic exercise, resistance exercise or multicomponent training to increase patients' physical activity. Despite the variations in intervention types, studies have demonstrated a correlation between exercise and the improvement of arterial stiffness in hypertensive and prehypertensive patients (47–49).

Aerobic exercise reduces PWV and improves arterial stiffness in hypertensive and prehypertensive patients. Increasing research suggests that aerobic exercise has a significant impact on blood pressure reduction in hypertensive and prehypertensive patients compared to the general population. Aerobic exercise reduces blood pressure in hypertensive and prehypertensive patients through various mechanisms, including reducing sympathetic nerve activity, increasing vagal tone, and improving left ventricular function and endothelial function (50). An increasing number of studies have begun to utilize aerobic exercise as a primary treatment for hypertensive and prehypertensive patients, as this intervention results in blood pressure reductions similar to those achieved with antihypertensive medication (51).

Oxidative stress and inflammation are the primary causes of vascular wall sclerosis (52, 53). Continuous exposed of the vascular to free radicals and inflammatory cytokines can lead to elastin fragmentation, collagen deposition, and smooth muscle proliferation (54). In addition, atherosclerosis is exacerbated by a decrease in nitric oxide (NO) and an increase in vasoconstrictors such as angiotensin, endothelin, and prostaglandins (55). Previous studies have shown that exercise is beneficial in remodelling and improving vascular compliance (56). Arteries in physically active individuals have higher elastin content and lower collagen content compared to those in inactive individuals (57). Furthermore, clinical studies have demonstrated that regular exercise exerts an anti-inflammatory effect by increasing anti-inflammatory cytokines [interleukin 4 (IL-4) and interleukin 10 (IL-10)] and decreasing pro-inflammatory cytokines [interleukin 6 (IL-6) and tumour necrosis factor alpha (TNF-α)] (58). Finally, there is evidence that exercise promotes NO production and decreases the concentration of vasoconstrictors such as endothelin I and angiotensin II (59).

### Subgroup analysis

In the studies we included, the exercise intervention significantly improved PWV in hypertensive and prehypertensive patients, yet there was considerable heterogeneity among the groups. Therefore, we employed subgroup analysis to interpret the results. In these subgroup analyses, we aimed to discern the effects of factors such as the type of exercise, aerobic exercise duration, intensity, frequency, session duration, weekly time, and subjects' age.

The Guidelines on Physical Activity and Sedentary Behaviour released by the World Health Organization (WHO) emphasize that everyone should engage in physical activity regardless of age or ability, as all forms of activity bring benefits. Regular physical activity is crucial for preventing cardiovascular diseases (CVDs). Aerobic exercise has a dose-response relationship in terms of health benefits, and the appropriate intensity of exercise is paramount (60). A previous study has highlighted the importance of conducting cardiorespiratory fitness testing before commencing exercise to determine the optimal exercise intensity (61). Current research has concluded that aerobic exercise is the most effective form of exercise for preventing and improving atherosclerosis (62), and that hypertensive and prehypertensive patients can significantly reduce systolic and diastolic blood pressure through aerobic exercise (63). Our study found that moderate-intensity aerobic exercise had a significant effect on improving PWV in hypertensive and prehypertensive patients, while vigorous-intensity aerobic exercise and multicomponent training did not show significant effects. This is consistent with the findings of Li et al. (64), showing that moderate-intensity aerobic exercise is more effective than other exercise types in improving arterial stiffness in hypertensive patients. Numerous studies have recommended aerobic exercise as a targeted treatment for hypertension, particularly for patients resistant to medication (65). Kohno et al. (66) found a significant drop in blood pressure in hypertensive patients after 3 weeks of moderate-intensity aerobic exercise. In addition, Zhang et al. (67) showed that 1 to 4 weeks of moderate-intensity training resulted in significant reductions in blood pressure and angiotensin secretion, and those changes were sustained through continued exercise. Furthermore, Donley et al. (68) also reported that aerobic exercise had a positive effect on improving arterial stiffness in hypertensive patients. An earlier meta-analysis showed that aerobic exercise could lower systolic and diastolic blood pressure in hypertensive patients by reducing vascular resistance and inhibiting the sympathetic nervous system and the adrenergic-angiotensin system (69). Moreover, weight loss leads to a decrease in plasma renin activity and aldosterone levels, resulting in a reduction in extracellular volume, a decline in sympathetic nervous system activity, and an improvement in insulin resistance. Exercise has been proven to be effective in altering body composition and reducing body weight (70–73). Existing studies have shown that regular aerobic exercise increases blood flow and exerts a higher shear stress on endothelial cells, thereby promoting the phosphorylation of endothelial-type NO synthase and the production of NO. This positively affects arterial stiffness through a series of signal transduction processes (74).

Our results showed that 12 or more weeks of aerobic exercise significantly improved PWV, while less than 12 weeks of aerobic exercise had no significant effect on improving PWV in hypertensive and prehypertensive patients. This effect typically manifests as a significant improvement in the early stages of the intervention, especially among patient groups that regularly engage in exercise. During these initial stages, exercise enhances vascular compliance and arterial elasticity through mechanisms such as reducing sympathetic nerve activity, increasing vagal tone, and improving endothelial function (75). However, there exists a certain dose-response relationship between the duration of the intervention and the sustainability of its effects. Previous studies have shown that the initial beneficial effect of exercise on improving arterial stiffness in hypertensive patients may fade over time (76). While cfPWV was reduced by 14%–23% after 3 months of aerobic exercise intervention, the reduction in cfPWV was not maintained if the intervention continued for 6 months (77). In addition, chronic low-grade inflammation, particularly involving IL-6, interleukin 8 (IL-8), and TNF-α, is considered to be associated with the pathogenesis of hypertension and atherosclerosis (78). Chronic exercise intervention may be impacted by chronic inflammation. Specifically, in hypertensive and prehypertensive patients, inflammatory responses (such as increased levels of IL-6 and TNF-α) are closely associated with the progression of atherosclerosis (79). While exercise can mitigate these inflammatory responses to a certain extent, overtraining or inappropriate high-intensity exercise may exacerbate them, thereby affecting the sustainability of the exercise benefits. Donley et al. (68) observed elevated levels of these inflammatory markers in hypertensive patients when comparing the experimental group with the control group. However, short-term exercise interventions did not induce any changes in the inflammatory markers in the subjects (80, 81). Periodic exercise typically leads to two adaptations: an increase in muscle glycogen content at rest and an enhanced ability of muscle to oxidize fat (82). Both of these adaptations are associated with a decrease in IL-6 (83). Conversely, exercise lasting more than 12 weeks for hypertensive patients can increase levels of IL-10, an anti-inflammatory and regulatory cytokine that controls indicators of chronic inflammation by inhibiting cells such as T and B lymphocytes and macrophages (84, 85). It is proposed that the improvement in vascular endothelial function during exercise is mediated by rapid alternations in cell signalling (86), whereas modifications in arterial stiffness involve long-term remodelling of the arterial wall's extracellular matrix. Generally, aerobic or other forms of exercise interventions require a minimum duration of 3 months or longer to improve arterial stiffness (87). On the other hand, mechanical stress induced by hypertension destroys elastin, promotes collagen deposition and fibrosis, leading to a gradual increase in atherosclerosis. Therefore, the improvement of endothelial function through exercise is limited, and such chronic atherosclerosis may take longer to improve, sometimes even becoming irreversible (88).

With regard to the frequency of intervention, our findings indicated that aerobic exercise conducted for three or more times per week significantly improved PWV, whereas aerobic exercise conducted for less than three times per week had no significant effect on PWV in hypertensive and prehypertensive patients. This is consistent with the findings of Guimarães et al. (34), showing that due to the specific characteristics of hypertensive patients, such as accelerated degradation of the elastic matrix, endothelial dysfunction, smooth muscle cell hypertrophy and proliferation, and an increase in collagen content, the improvement of arterial stiffness in these patients requires an increase in the intensity or frequency of interventions. In a study by Hansen et al. (89), a 12-week intervention program involving three weekly sessions of aerobic exercise for 48 hypertensive male and female patients resulted in a significant reduction in SBP and DBP. Similar to our findings, Sosner et al. (42, 90) observed a decrease in mean heart rate in subjects who trained three times per week, potentially indicating improved myocardial autoregulation along with a significant increase in cardiac vagal tone.

Regarding the session duration, our findings indicated that aerobic exercise conducted for up to 60 min was more effective in improving PWV in hypertensive and prehypertensive patients. A previous study has demonstrated a dose-effect relationship between exercise and health, emphasizing that appropriate exercise load is crucial for promoting health (60). Excessive exercise duration does not yield positive health benefits and may even have adverse effects. Cai et al. (91) showed that engaging in 45–60 min of exercise three times per week was beneficial for improving the health of older adults. In addition, previous studies have shown that exercising for 20 min at a time can positively affect the health of the organism. However, too short a period of exercise does not lead to improvements in brain structure and function, while excessive exercise duration can result in fatigue, diminishing the effectiveness of exercise interventions (60, 92).

WHO recommends that all adults, including those with chronic illnesses or disabilities, should engage in at least 150 to 300 min of moderate- to vigorous-intensity aerobic activity weekly, and children and adolescents should average 60 min per day. Simultaneously, WHO emphasizes that this recommendation also applies to older adults. Our subgroup analyses indicated that aerobic exercise conducted for 180 or more min per week was more effective in improving PWV in hypertensive patients. Atherosclerosis mechanisms include elastic matrix degradation, endothelial dysfunction, smooth muscle cell hypertrophy and proliferation, and increased collagen content, which occur more rapidly in hypertensive patients (93). In this context, as observed in the study by Guimarães et al. (34), aerobic exercise of longer duration may be necessary to improve the degree of atherosclerosis in hypertensive patients. To achieve the exercise duration of 180 min or more per week, as mentioned above, moderate-intensity aerobic exercise with session duration of 60 min or less and an intervention frequency of three or more times per week is more beneficial for improving arterial stiffness in hypertensive and prehypertensive patients. Therefore, for hypertensive and prehypertensive patients, the recommended exercise pattern should involve reducing the session duration and increasing the weekly exercise frequency to meet the exercise duration of 180 min or more per week.

Regarding the age of the subjects, our findings suggested that aerobic exercise was more effective in improving PWV in middle-aged hypertensive and prehypertensive patients. As the body inevitably ages, aerobic exercise has a limited effect on improving arterial stiffness. As individuals age, there is a gradual decline in the elasticity of blood vessels, primarily due to a decrease in elastin and an increase in collagen deposition. This phenomenon contributes to the reduced responsiveness of older adult individuals to exercise interventions compared to younger individuals (94). While exercise can enhance certain physiological indicators, the older adult population often finds it difficult to achieve substantial improvements due to prolonged vascular sclerosis and the impact of chronic diseases (79). Furthermore, the older adult population frequently exhibits elevated levels of oxidative stress and inflammation, which is closely associated with atherosclerosis and may potentially negate the beneficial effects of exercise. Ha et al. (95) found that 12 weeks of aerobic exercise did not improve PWV in females aged 70–80 years, which may be related to the decreased levels of sex hormones in the older adult. In addition, a previous study has reported an age-related decline in sex hormones, with a sharp drop in sex hormone levels after the age of 65 (96). Sex hormone levels affect the effects of aerobic exercise on the improvement of cardiovascular function (97). Furthermore, aortic PWV increases by approximately 0.10 m/s per year with age, significantly increasing the incidence of atherosclerosis in the older adult (93). A study by Madden et al. (36) found that aerobic exercise significantly reduces PWV in both healthy and diseased individuals, and the healthier the individual, the greater the reduction. However, due to aging, the probability of CVDs increase in older adult individuals, and aerobic exercise had no significant effect on atherosclerosis in older adults with cardiometabolic risk factors. An additional factor to be considered is the discrepancy in exercise capacity among older adult individuals. Research has demonstrated that moderate-intensity aerobic exercise has a substantial impact on reducing PWV. However, given their constrained exercise tolerance, older adult individuals may encounter challenges in sustaining moderate-intensity aerobic exercise for extended periods (30). Consequently, when devising an exercise intervention strategy for older adult patients, it is imperative to take their exercise capacity and tolerance into account to ensure the efficacy of the intervention.

### Heterogeneity analysis

The type of intervention was identified as a key factor contributing to the observed heterogeneity among the studies included. The studies employed a range of exercise interventions, including aerobic exercise, resistance training, and mixed training modalities. These exercise modalities are hypothesized to exert distinct effects on PWV (98). Aerobic exercise has been shown to continuously increase shear stress and consequently reduce PWV by enhancing endothelial function. In contrast, resistance training has been observed to produce a different effect by inducing intermittent increases in NO synthesis (comparable to ischemia-reperfusion) (75). Consequently, the type of the intervention may exert a substantial influence on the outcomes observed.

Furthermore, there is considerable heterogeneity in the duration, frequency, and intensity of exercise interventions across studies. For instance, some studies employed a relatively brief intervention period (e.g., less than 12 weeks), while others assessed training over a more extended duration (e.g., 12 weeks or more). A limited intervention period may not demonstrate a substantial effect of exercise on PWV, thereby introducing heterogeneity among the studies (99). Conversely, the intensity and frequency of exercise regimens, such as moderate to high-intensity training performed three times per week or more, have been shown to significantly impact the intervention effect (99, 100).

Beyond the discrepancies inherent to exercise interventions, the age of subjects, their health status (e.g., the presence of concomitant complications or underlying diseases), and the severity of hypertension are pivotal factors contributing to the heterogeneity of studies. As individuals age, improving cardiovascular function becomes increasingly challenging, and the efficacy of exercise interventions may be comparatively diminished among the older adult (101). The decline in physical capacity with age can limit the feasibility of certain exercises, particularly those involving greater intensity, duration, or complexity. The elasticity of blood vessels decreases with age, resulting in stiffer and thinner vessel walls, which can also diminish the efficacy of exercise interventions in addressing atherosclerosis. Furthermore, heightened levels of oxidative stress and chronic inflammation may serve as additional inhibitors of the impact of exercise interventions. These physiological factors may be the primary contributors to the observed discrepancies in study findings.

### Strengths and limitations of this systematic review

In this systematic review and meta-analysis, we investigated the optimal modalities that can improve arterial stiffness in hypertensive and prehypertensive patients. Our findings provide an optimal pattern of exercise pattern that can improve arterial stiffness in hypertensive and prehypertensive patients. Clinically, hypertensive and prehypertensive patients, particular those in middle age, can improve their arterial stiffness by engaging in moderate-intensity aerobic exercise for at least 12 weeks, with session duration lasting no more than 60 min and occurring more than 3 times per week, totaling over 180 min of exercise per week.

These specific parameters for aerobic exercise represent a novel contribution to the existing literature, as they provide a clear and actionable guidance for clinical practitioners and patients alike. By adhering to this optimal exercise pattern, hypertensive and prehypertensive patients can potentially reduce their risk of cardiovascular disease and improve their overall health outcomes. In summary, our study not only confirms the benefits of aerobic exercise in improving arterial stiffness but also provides specific, evidence-based recommendations for exercise modality, duration, and frequency. These novel findings have the potential to significantly impact clinical practice and patient care.

However, this study has some potential limitations. Due to constrains such as sample size and data availability, we focused primarily on aerobic exercise, leaving resistance exercise unanalyzed. Therefore, we were unable to examine the effects of these training modalities on arterial stiffness in hypertensive and prehypertensive patients. In addition, complete blinding in study inclusion was not feasible, leading to potential subjective biases in the quality assessment process. Moreover, the variety of exercise types included in our studies, such as Pilates and taekwondo, prevented us from determining the most beneficial type for improving arterial stiffness in hypertensive and prehypertensive patients. Finally, with the advancement of technology, digital interventions are increasingly being applied to enhance patient compliance and monitoring. Therefore, future research can further explore the application of digital interventions in improving endothelial function in hypertensive and prehypertensive patients.

## Conclusion

Exercise improved PWV in hypertensive and prehypertensive patients. To improve arterial stiffness, this meta-analysis provides clinicians with evidence to recommend that hypertensive and prehypertensive patients, particularly middle-aged individuals, participate in a minimum of 12 weeks of moderate-intensity aerobic exercise at least 3 times per week for less than 60 min per session, with a goal of 180 min per week being achieved by increasing the frequency of exercise.

## Data Availability

The original contributions presented in the study are included in the article/Supplementary Material, further inquiries can be directed to the corresponding author/s.
