# Supplementary material for: Effects of exercise on pulse wave velocity in hypertensive and prehypertensive patients: a systematic review and meta-analysis of randomized controlled trials
Source: Front Cardiovasc Med. 2025 Feb 17;12:1504632. doi: 10.3389/fcvm.2025.1504632 (PMC11872916; doi:10.3389/fcvm.2025.1504632)
Supplement: Supplementary file 1 [file Datasheet1.pdf]

Supplementary Material

1 Supplementary Figures and Tables

1.1 Supplementary Figures

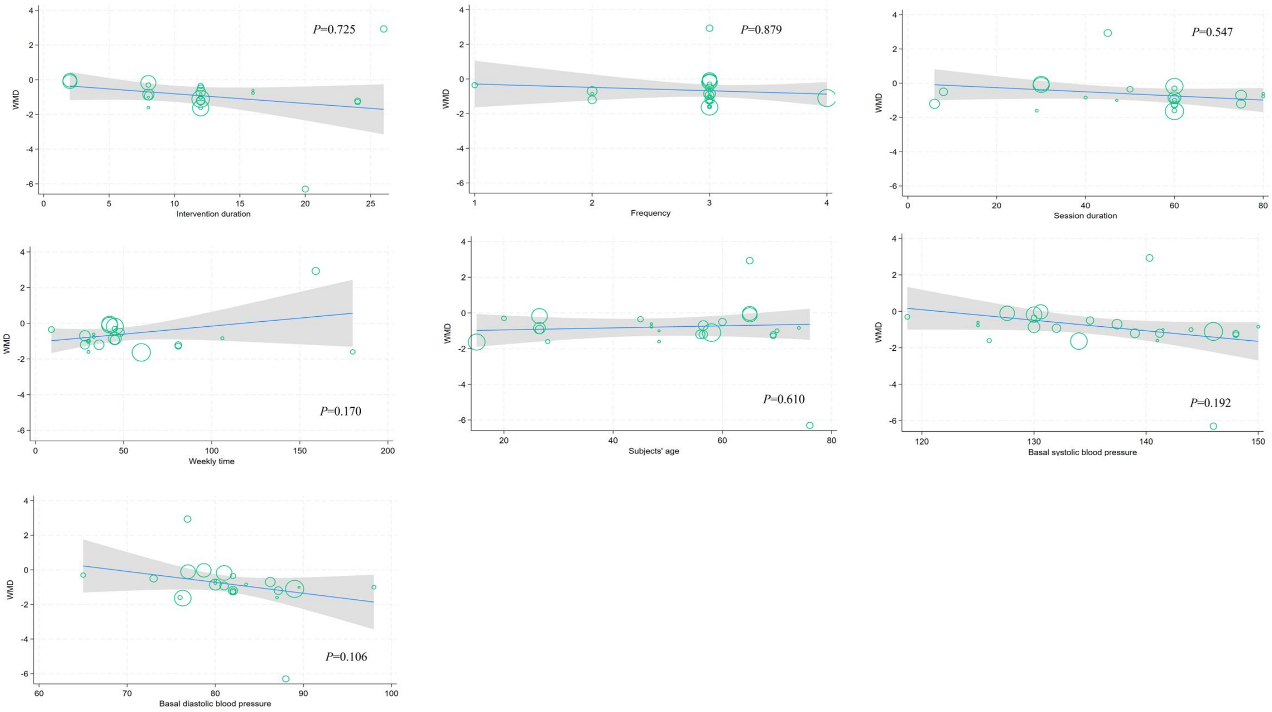

Supplementary Figure 1. Meta-regression analysis results.

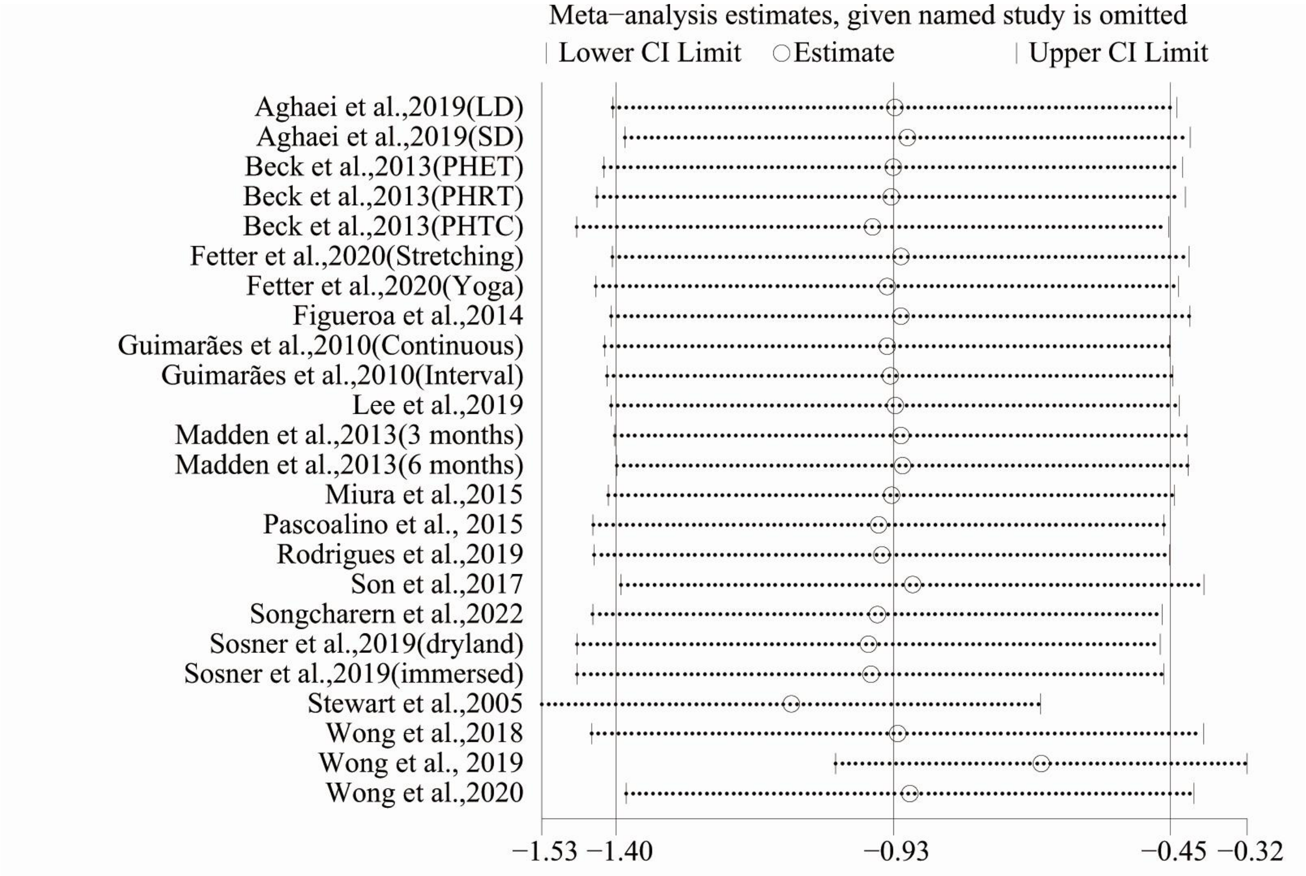

Supplementary Figure 2. Sensitivity analysis results.

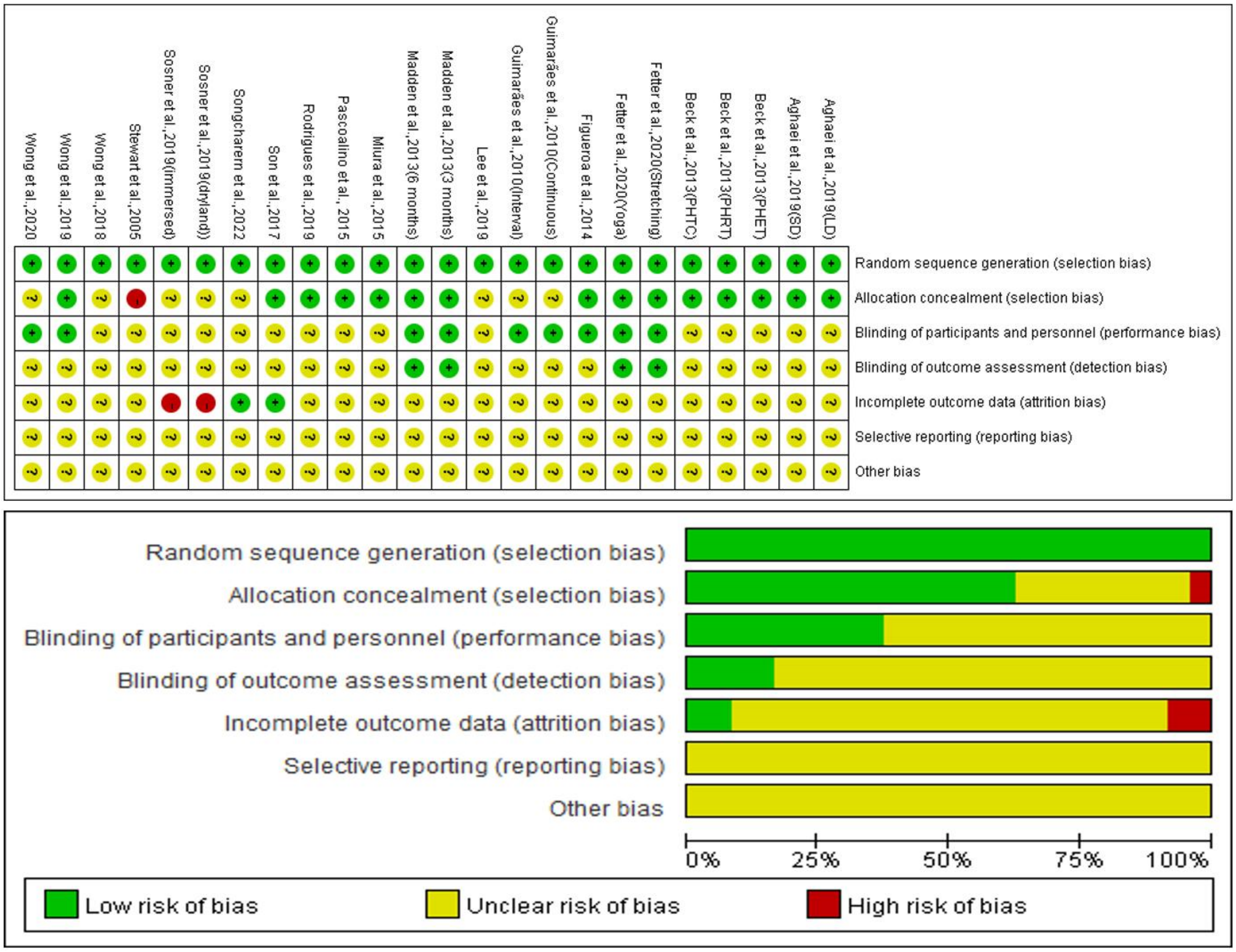

Supplementary Figure 3. Results of Cochrane risk of bias tool.

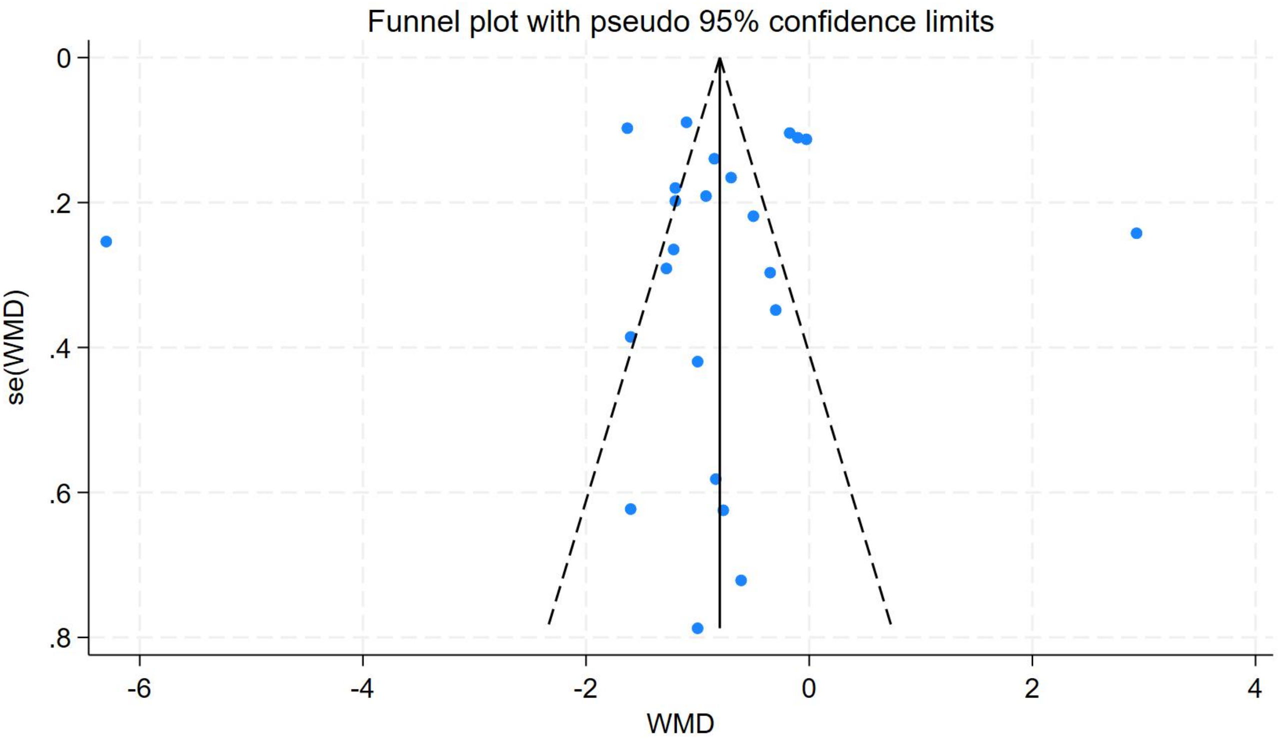

Supplementary Figure 4. Funnel plot.

1.2 Supplementary Tables

Supplementary Table 1. Methodological assessment of randomized controlled trials included in the systematic review using the PEDro scale

Study	A	B	C	D	E	F	G	H	I	J	K	Score
Aghaei et al., 2019	Y	1	1	1	0	0	0	1	1	1	1	7
Beck et al., 2013	Y	1	1	1	1	0	1	1	1	1	1	9
Fetter et al., 2020	Y	1	1	1	1	0	0	0	1	1	1	7
Figueroa et al., 2014	Y	1	1	1	1	1	1	0	1	1	1	9
Guimaraes et al., 2010	Y	1	1	1	1	1	1	0	1	1	1	9
Lee et al., 2019	Y	1	1	1	1	0	0	1	1	1	1	8
Madden et al., 2013	Y	1	1	1	1	0	0	1	1	1	1	8
Miura et al., 2015	Y	1	1	1	1	0	0	0	1	1	1	7
Pascoalino et al., 2015	Y	1	1	1	1	0	0	1	1	1	1	8
Rodrigues et al., 2019	Y	1	1	1	1	0	0	1	0	1	1	7
Son et al., 2017	Y	1	1	1	1	0	0	1	1	1	1	8
Songcharern et al., 2022	Y	1	1	1	1	0	0	1	1	1	1	8
Sosner et al., 2019	Y	1	1	1	1	0	0	1	1	1	1	8
Stewart et al., 2005	Y	1	1	1	1	0	0	1	1	1	1	8
Wong et al., 2018	Y	1	1	1	1	0	0	1	1	1	1	8
Wong et al., 2019	Y	1	1	1	1	1	1	1	1	1	1	10

Wong et al., 2020	Y	1	1	1	1	1	1	1	1	1	1	10

A, eligibility criteria; B, random allocation; C, concealed allocation; D, baseline comparability; E, blind subjects; F, blind therapists; G, blind assessors; H, adequate follow-up; I, intention-to-treat analysis; J, between-group comparisons; K, point estimates and variability. The total score represents the score of the PEDro scale. Item A was not scored. Y: yes

Table S2. Results of Egger's test

Std_EFF	Coef.	Std. Err.	t	P > |t|	95%CI
Slope	-0.658	0.449	-1.47	0.157	-1.589, 0.273
Bias	-0.955	2.557	-0.37	0.712	-6.257, 4.347

Abbreviations: Coef, coefficient; Std. Err, standard error; t, t-test statistic; p, probability; 95% CI, 95% Confidence Interval.
